# Global burden of breast cancer and attributable risk factors in 195 countries and territories, from 1990 to 2017: results from the Global Burden of Disease Study 2017

**DOI:** 10.1186/s13045-019-0828-0

**Published:** 2019-12-21

**Authors:** Na Li, Yujiao Deng, Linghui Zhou, Tian Tian, Si Yang, Ying Wu, Yi Zheng, Zhen Zhai, Qian Hao, Dingli Song, Dai Zhang, Huafeng Kang, Zhijun Dai

**Affiliations:** 1 Department of Breast Surgery, The First Affiliated Hospital, School of Medicine, Zhejiang University, Hangzhou, 310003 China; 2 Department of Oncology, The Second Affiliated Hospital of Xi’an Jiaotong University, Xi’an, 710004 China

**Keywords:** Breast cancer, Global cancer burden, Disability-adjusted life years, Alcohol use, Incidence

## Abstract

**Background:**

Statistical data on the incidence, mortality, and burden of breast cancer and the relevant risk factors are valuable for policy-making. We aimed to estimate breast cancer incidence, deaths, and disability-adjusted life years (DALYs) by country, gender, age group, and social-demographic status between 1990 and 2017.

**Methods:**

We extracted breast cancer data from the 2017 Global Burden of Disease (GBD) study from 1990 through 2017 in 195 countries and territories. Data about the number of breast cancer incident cases, deaths, DALYs, and the age-standardized rates were collected. We also estimated the risk factors attributable to breast cancer deaths and DALYs using the comparative risk assessment framework of the GBD study.

**Results:**

In 2017, the global incidence of breast cancer increased to 1,960,681 cases. The high social-development index (SDI) quintile included the highest number of breast cancer death cases. Between 2007 and 2017, the ASDR of breast cancer declined globally, especially in high SDI and high middle SDI countries. The related DALYs were 17,708,600 in 2017 with high middle SDI quintile as the highest contributor. Of the deaths and DALYs, alcohol use was the greatest contributor in most GBD regions and other contributors included high body mass index (BMI) and high fasting plasma glucose.

**Conclusion:**

The increasing global breast cancer burden is mainly observed in lower SDI countries; in higher SDI countries, the breast cancer burden tends to be relieving. Therefore, steps against attributable risk factors should be taken to reduce breast cancer burden in lower SDI countries.

## Background

Breast cancer was the third highest incident cancer in 2017, with an estimated 1,960,681 (95% UI = 1,891,447–2,023,170) incident cases and a high prevalence in females. Breast cancer is the leading cause of cancer death in females and also a non-negligible cause of cancer death in males worldwide, claiming 181,004 lives and resulting in 17.7 million disability-adjusted life years (DALYs), making it one of the most severe burdensome cancer globally [1, 2]. Breast cancer incidence and mortality are still increasing, both in developing and developed countries [3]. Although the survival rate in breast cancer has improved, it varies in different countries distinctly [4], due to factors such as lack of early-stage screening, detection, and cost-effective therapy [5]. To better understand the enormous influence of this disease on public health, it is worthwhile to review and analyze the related global trends.

Thus far, several regional and national studies on breast cancer incidence and mortality have been performed, and the results of these multifarious studies from different parts of the world present an inclusive picture. Epidemiological studies from Arab countries [6], India [7], Latin America [8], and Europe [9] show an alarmingly rising burden with associated incidence and mortality. However, specific studies of breast cancer burden at a global level are lacking. The aim of this study was to describe the influence of geographical location, social-development index (SDI), age, and gender on the global trends in the incident cases, deaths, and DALYs of breast cancer based on data from the Global Burden of Disease from 1990 to 2017 in 195 countries and territories.

## Materials and methods

### Study data

Data for the disease burden of breast cancer were obtained from an online data source tool, the Global Health Data Exchange (GHDx) query tool (http://ghdx.healthdata.org/gbd-results-tool), which is an ongoing global collaboration that uses all available epidemiological data to provide a comparative assessment of health loss from 328 diseases across 195 countries and territories. From GBD study 2017, we obtained data on annual incidence, death, DALY, and respective age-standardized rate (ASR) of breast cancer from 1990 to 2017. The SDI, which is based on national-level income per capita, average years of education among persons older than 15, and total fertility rate, was used to categorize the countries into five SDI quintiles (high, high-medium, medium, low-medium, and low levels).

### Estimation framework

Estimation of cancer incidence was based on individual cancer registries or integrated databases of cancer registries. Systematic literature search was performed in PubMed to find the evidence for breast cancer deaths due to the attributable risk factors (alcohol use, high body mass index, high fasting plasma glucose, low physical activity, smoking, and secondhand smoke). For each included study, the proportions of breast cancer cases induced by the specific risk factors were calculated. The proportion data obtained from systematic literature review were applied to four independent DisMod-MR2.1 as inputs [10]. Mortality data of breast cancer from vital registration systems and mortality estimates were used as input data into the CODEm (Cause of Death Ensemble Model) [11]. The CODEm predicts mortality based on available data and covariates such as education, smoking, SDI, lagged distributive income, and alcohol use. Single cause estimates were adjusted to fit for the separately estimated all-cause mortality using the Cod-Correct algorithm [12, 13].

Years lived with disability (YLDs) were calculated by multiplying the prevalence of each sequela by its disability weight and by adding the procedure-related morbidity associated with breast cancer treatment. YLLs due to breast cancer were calculated using standard global life expectancy and the number of deaths according to age [12]. Breast cancer DALYs were calculated as the sum of YLDs and YLLs.

### Attributable burden

The GBD study incorporated the comparative risk assessment framework previously [14] to quantify the burden of several causes and impairments attributable to 84 environmental, occupational, metabolic, and behavioral risk factors. Briefly, after assessing the casual evidence in each risk-outcome pair, we selected 2 components to model the attributable burden of causes to risks, including deaths and DALYs.

### Statistical analysis

ASRs, DALYs, and the estimated annual percentage changes (EAPCs) were calculated to quantify breast cancer incidence and mortality trends. ASRs (per 100,000 population) were calculated on the basis of the following formula [15].

$$ \mathrm{ASR}=\frac{\sum \limits_{i=1}^A{a}_i{w}_i}{\sum \limits_{i=1}^A{w}_i}\times 100,000, $$

(*a*_*i*_, where *i* denotes the *i*th age class, and the number of persons (or weight) (w_*i*_) in the same age subgroup *i* of the selected reference standard population.)

Moreover, trends in ASIR reflect the alterations in human disease patterns and risk factors. The concept of EAPC is introduced to describe the trends in ASR within a specified time interval, as it is assumed that the natural logarithm of ASR is linear along with time. Thus, *Y* = *α*+*βX*+*ε*, where *Y* refers to ln(ASR), *X* represents calendar year, and *ε* represents error term. Based on this formula, *β* determines the positive or negative trends of ASR. The formula for calculating EAPC is EAPC = 100 × (exp(*β*) − 1) and 95% confidence intervals are obtained from the linear model [16, 17]. It is shown that when EAPC and the lower boundary of the confidence interval are positive, then ASR is in an upward trend. Conversely, when EAPC and the upper boundary of the confidence interval are negative, the ASR is in a descending trend. All statistical analyses were performed using the R program (Version 3.5.2, R core team). A *p* value of less than 0.05 was considered statistically significant.

## Results

### Breast cancer incidence burden

**Fig. 1:**
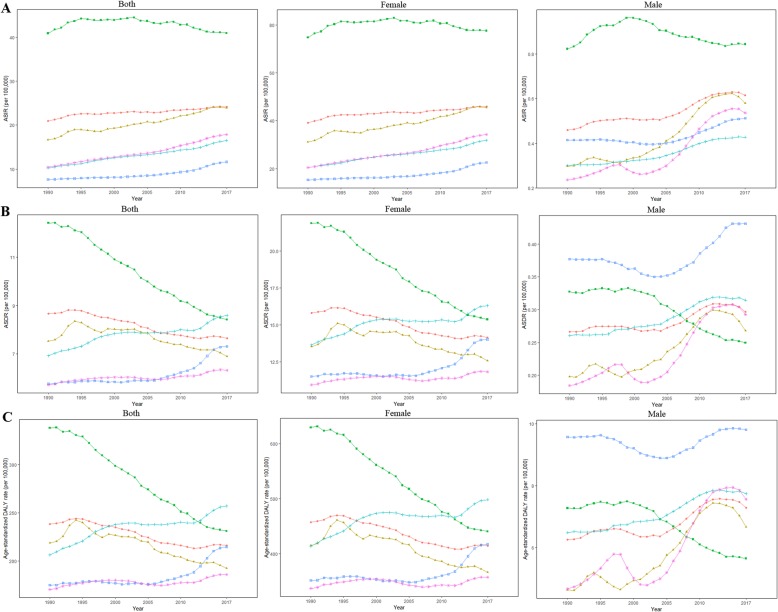
Age-standardized breast cancer incidence (**a**), death (**b**), and DALY (**c**) rates globally (red lines with circles), and in social-demographic index high (green lines with squares), high middle (yellow lines with triangles), middle (purple lines with snowflakes), low middle (baby-blue lines with “+”), and low (navy-blue lines with hollow squares) quintiles

Globally, the incident cases of breast cancer has increased to 1,960,681(95% UI = 1,891,447–2,023,170) with an ASIR of 24.19/100,000 persons (95% UI = 23.34–24.86) in 2017 (Table 1). There were 16,697,282 breast cancer patients (95% UI = 16,178,870–17,171,735) in 2017 globally. Alarmingly, the number of breast cancer incident cases increased in all SDI quintiles between 1990 and 2017, precipitously increased in middle SDI and low middle SDI countries (2.62-fold and 2.28-fold, respectively) and less obviously in high SDI countries (0.57-fold). The ASIR of breast cancer increased the fastest in low SDI, while it decreased in high SDI quintile between 2007 and 2017 (Fig. 1a).

**Table 1:** The incidence cases and age-standardized incidence of breast cancer in 1990 and 2017, and its temporal trends from 1990 to 2017

Characteristics	1990		2017		1990-2017
	Incidence cases No. × 10^3^ (95% UI)	ASR per 100,000 No. (95% UI)	Incidence cases No. × 10^3^ (95% UI)	ASR per 100,000 No. (95% UI)	EAPC No. (95% CI)
Overall	878.7 (846.0–935.7)	20.91 (20.18–22.16)	1960.7 (1891.4–2023.2)	24.19 (23.34–24.96)	0.42 (0.35–0.47)
Sex					
Male	8.5 (8.2–8.9)	0.46 (0.44–0.48)	23.1 (22.3–24.0)	0.61 (0.59–0.64)	1.17 (1.01–1.34)
Female	870.2 (837.7–927.1)	39.19 (37.77–41.66)	1937.6 (1868.0–2000.4)	45.91 (44.24–47.40)	0.45 (0.38–0.51)
Social-demographic index					
Low	30.0 (23.6–42.0)	7.61 (6.10–10.53)	94.4 (86.9–102.7)	11.62 (10.70–12.70)	1.44 (1.22–1.67)
Low middle	67.8 (56.5–89.4)	10.25 (8.62–13.33)	222.6 (199.3–275.6)	16.50 (17.75–20.62)	1.65 (1.58–1.72)
Middle	115.6 (106.6–130.0)	10.43 (9.71–11.66)	418.9 (378.2–444.1)	17.86 (16.12–18.92)	1.95 (1.87–2.03)
Middle high	165.7 (157.8–177.6)	16.63 (15.86–17.82)	436.0 (400.4–458.7)	23.93 (22.00–25.15)	1.27 (1.17–1.37)
High	497.4 (491.8–503.3)	40.92 (40.47–41.41)	781.3 (757.5–804.4)	40.99 (39.76–42.21)	− 0.13 (− 0.25–0.01)
Region					
Central Asia	7.7 (7.4–8.0)	14.90 (14.43–15.40)	16.6 (15.5–17.8)	19.66 (18.43–21.00)	1.06 (1.00–1.12)
Central Europe	36.6 (35.6–37.7)	24.92 (24.25–25.62)	60.4 (57.6–63.4)	32.05 (30.60–33.70)	0.97 (0.85–1.08)
Eastern Europe	63.1 (60.4–65.9)	22.97 (21.93–23.98)	97.2 (93.5–100.8)	30.64 (29.43–31.85)	0.82 (0.56–1.08)
Australasia	9.7 (9.4–10.0)	42.18 (40.84–43.65)	18.8 (16.4–21.4)	44.22 (38.38–50.73)	− 0.10 (− 0.29–0.09)
High-income Asia Pacific	34.0 (33.0–35.1)	16.35 (15.87–16.85)	91.9 (85.8–97.8)	27.03 (25.31–28.75)	2.32 (2.02–2.62)
High-income North America	198.2 (195.0–201.4)	58.43 (57.51–59.38)	276.9 (266.9–287.8)	49.34 (47.62–51.52)	− 1.04 (− 1.20–0.88)
Southern Latin America	10.9 (10.5–11.3)	23.21 (22.36–24.14)	22.7 (20.2–25.5)	28.78 (25.69–32.45)	0.62 (0.45–0.80)
Western Europe	233.9 (229.9–237.9)	44.13 (43.39–44.92)	341.8 (324.9–358.9)	45.41 (43.21–47.65)	0.02 (− 0.14–0.19)
Andean Latin America	2.1 (1.9–2.4)	9.01 (8.19–10.07)	8.3 (7.3–9.6)	14.77 (12.92–17.07)	1.82 (1.62–2.02)
Caribbean	5.5 (5.1–6.0)	20.06 (18.74–21.94)	14.2 (12.6–15.9)	27.85 (24.80–31.29)	1.16 (1.09–1.23)
Central Latin America	13.2 (12.9–13.5)	13.35 (13.06–13.67)	51.4 (48.7–54.2)	21.09 (20.01–22.21)	1.49 (1.35–1.64)
Tropical Latin America	16.4 (16.0–16.9)	15.69 (15.31–16.07)	54.4 (52.5–56.2)	22.52 (21.79–23.28)	1.15 (0.89–1.42)
North Africa and Middle East	20.3 (16.7–27.7)	9.77 (8.15–13.21)	91.2 (85.2–100.6)	18.06 (16.90–20.28)	2.49 (2.27–2.70)
South Asia	53.7 (46.7–67.3)	7.61 (6.70–9.47)	210.8 (182.3–248.9)	14.07 (12.17–16.61)	2.03 (1.81–2.25)
East Asia	99.0 (89.5–120.5)	9.43 (8.55–11.52)	386.5 (326.1–417.0)	18.41 (15.49–19.86)	2.63 (2.42–2.85)
Oceania	0.6 (0.5–0.9)	16.28 (13.49–22.11)	1.7 (1.3–2.4)	20.15 (15.82–26.69)	0.86 (0.83–0.89)
Southeast Asia	41.7 (34.5–51.6)	13.04 (10.98–15.92)	125.6 (115.2–135.9)	18.70 (17.22-20.19)	1.30 (1.23–1.36)
Central Sub-Saharan Africa	2.7 (1.9–4.2)	9.87 (7.37–14.73)	8.1 (6.2–10.7)	13.32 (10.70–17.01)	0.99 (0.90–1.08)
Eastern Sub-Saharan Africa	10.4 (8.0–14.3)	11.17 (8.85–15.22)	25.6 (22.3–29.5)	12.9 (11.4–14.8)	0.27 (0.09–0.44)
Southern Sub-Saharan Africa	4.2 (3.9–4.6)	13.23 (12.07–14.67)	10.5 (9.5–11.4)	17.44 (15.75–18.82)	1.02 (0.49–1.55)
Western Sub-Saharan Africa	14.6 (10.2–20.8)	14.41 (10.11–20.28)	46.1 (35.1–61.0)	20.57 (15.80–26.95)	1.34 (1.22–1.46)

*ASR* age-standardized rate, *CI* confidence interval, *EAPC* estimated annual percentage change, *UI* uncertainty interval

**Fig. 2:**
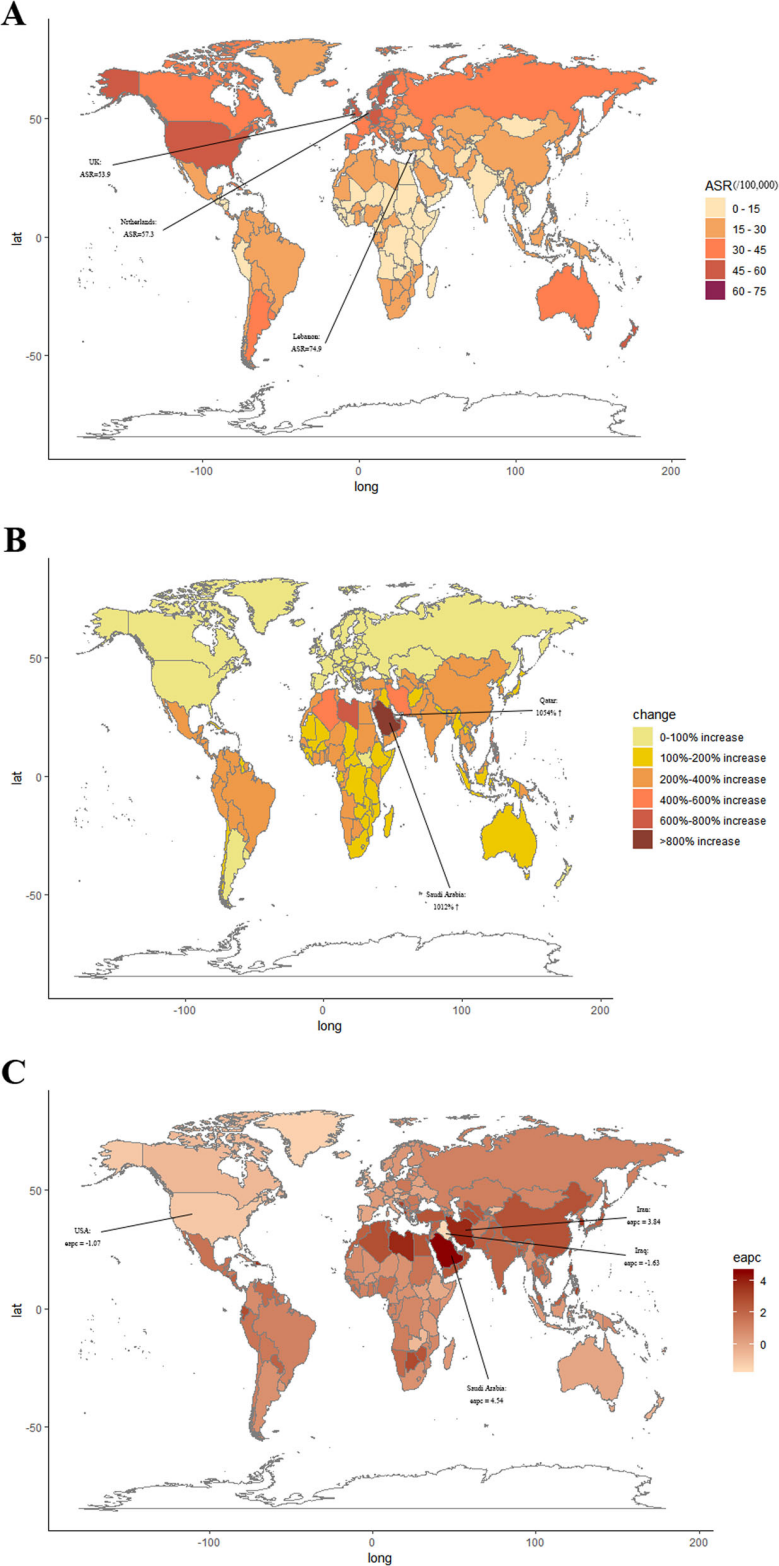
The global disease burden of breast cancer for both genders in 195 countries and territories. **a** The ASR of breast cancer in 2017. **b** The relative incidences changes of breast cancer between 1990 and 2017. **c** The EAPC of breast cancer ASR from 1990 to 2017. Countries with an extreme number of cases or changes were lined out. ASR, age-standardized rate; EAPC, estimated annual percentage change

Regionally, the incidences increased in all regions between 1990 and 2017 with the largest increases in North Africa and the Middle East and the lowest increases in high-income regions like North America, Western Europe, and Eastern Europe (Table 1). Only in high-income North America, the ASIR of breast cancer was found to have decreased. China, the USA, and India were the 3 countries with the highest reported new cases of breast cancer in 2017 while Marshall Islands, Greenland, and Kiribati were the 3 countries with the least. Lebanon, the Netherlands, and the UK showed the highest ASIR while Niger, Malawi, and Sudan showed the lowest ASIR in 2017 (Fig. 2a and Additional file 1: Figure S1).

### Breast cancer deaths and DALY burden

Globally, the high SDI quintile had the highest number of breast cancer deaths (181,004, 95% UI = 176,078–186,127) in 2017. However, in the decennium between 2007 and 2017, the ASDR of breast cancer has declined globally, especially in the high SDI and the high middle SDI quintiles (Table 2 and Fig. 1b). Meanwhile, breast cancer-related DALYs were 17,708,600 (95% UI = 16,899,498–18,674,972) for which the high middle SDI quintile contributed the most. The total DALYs increased in all SDI quintiles with the highest increase in the low SDI quintile and the lowest increase in the high SDI quintile. Correspondingly, the age-standardized DALY rate decreased most seriously in high SDI quintile and most slightly in the low SDI quintile (Table 2 and Fig. 1c).

**Table 2:** Breast cancer death cases, DALYs, and related age-standardized rates

Region metric	All ages × 10^3^ (95% UI)				Age-standardized rate/100,000 (95% UI)			
	2017 no. male + female	2017 no. male	2017 no. female	% 1990–2017 change	2017 male + female	2017 male	2017 female	% 1990–2017 change
Global								
Deaths	611.6 (589.2–640.7)	10.9 (10.5–11.4)	600.7 (578.7–629.9)	74.96 (57.34–87.02)	7.65 (7.37–8.01)	0.30 (0.29–0.31)	14.14 (13.63–14.84)	− 11.66 (− 20.06–6.02)
DALYs	17708.6 (16899.5–18675.0)	285.5 (272.8–299.4)	17423.1 (16617.5–18378.2)	69.73 (49.96–84.04)	216.3 (206.4–228.1)	7.28 (6.96–7.64)	414.7 (395.5–437.6)	− 9.24(− 19.51–1.88)
High SDI								
Deaths	181.0 (176.1–186.1)	2.5 (2.4–2.6)	178.5 (173.6–183.6)	16.48 (13.05–19.68)	8.42 (8.18–8.64)	0.25 (0.24–0.26)	15.37 (14.94–15.78)	− 32.23 (− 34.22–30.42)
DALYs	4290.9 (4080.9–4514.7)	52.5 (49.8–55.8)	4238.4 (4030.9–4460.0)	5.63 (2.03–8.81)	231.2 (220.0–243.1)	5.65 (5.35–6.00)	440.6 (419.5–463.6)	− 31.67 (− 34.00–29.53)
High middle SDI								
Deaths	123.4 (114.3–128.2)	2.2 (2.1–2.3)	121.2 (112.1–126.0)	72.76 (46.19–84.08)	6.89 (6.38–7.16)	0.27 (0.25–0.28)	12.58 (11.65–13.09)	− 8.37(− 22.48–2.52)
DALYs	3536.3 (3259.6–3720.8)	58.2 (54.4–62.0)	3478.1 (3203.1–3663.6)	59.30 (34.20–71.26)	192.3 (177.0–202.4)	6.66 (6.23–7.09)	366.7 (337.5–386.3)	− 12.17(− 26.10–5.59)
Middle SDI								
Deaths	142.5 (128.5–150.4)	3.1 (2.9–3.3)	139.4 (125.5–147.4)	144.64 (96.48–172.78)	6.32 (5.70–6.67)	0.29 (0.27–0.31)	11.83 (10.64–12.50)	10.59 (− 11.08–22.71)
DALYs	4433.9 (3996.3–4723.9)	86.6 (80.5–92.8)	4347.3 (3909.3–4637.7)	126.21 (82.70–154.77)	185.9 (167.2–197.9)	7.56 (7.02–8.09)	357.4 (320.7–381.1)	9.12 (− 12.08–22.40)
Middle-low SDI								
Deaths	108.0 (96.0–137.0)	1.7 (1.6–1.9)	106.3 (94.3–135.2)	154.40 (85.42–218.23)	8.59 (7.62–10.99)	0.31 (0.29–0.33)	16.30 (14.44–20.89)	24.10 (− 8.76–52.76)
DALYs	3569.7 (3178.2–4459.9)	48.9 (45.6–52.2)	3520.8 (3131.0–4409.3)	149.61 (80.17–217.70)	257.2 (229.1–322.6)	7.74 (7.20–8.24)	498.1 (443.1–626.1)	24.63 (− 9.51–57.13)
Low SDI								
Deaths	54.8 (50.5–59.9)	1.4 (1.2–1.5)	53.5 (49.2–58.6)	159.35 (85.72–223.84)	7.31 (6.72–7.95)	0.43 (0.38–0.47)	14.02 (12.90–15.34)	26.93 (− 8.42–56.46)
DALYs	1819.8 (1680.3–1980.8)	37.7 (34.2–41.2)	1782.1 (1642.6–1943.8)	149.04 (77.91–217.13)	214.7 (198.4–233.7)	9.81 (8.83–10.70)	417.5 (385.0–455.5)	22.74 (− 12.23–54.50)

Regionally, a number of breast cancer deaths were the highest in South Asia during the study period, reaching 108,966 (95% UI = 93,488–131,457) cases in 2017. Meanwhile, South Asia showed the largest increase of breast cancer deaths between 1990 and 2017. Only in high-income North America, Western Europe, and Australasia, ASDR decreased. The countries with the largest populations, including China, India, and the USA, had the most deaths in 2017 while the ASDR was highest in Fiji, Tonga, and the Bahamas (Fig. 2b). South Asia, East Asia, and Western Europe were the areas with the highest breast cancer-related DALYs in 2017. Among the 21 GBD regions, only Western Europe showed a declined trend of DALY cases. The age-standardized DALY rate increased in 12 regions, while it decreased in 9 GBD regions. The highest number of DALY cases was observed in China, India, and the USA, whereas the Bahamas, Nigeria, and Tonga were the 3 countries with the highest age-standardized DALY rate (Fig. 2c).

**Fig. 3:**
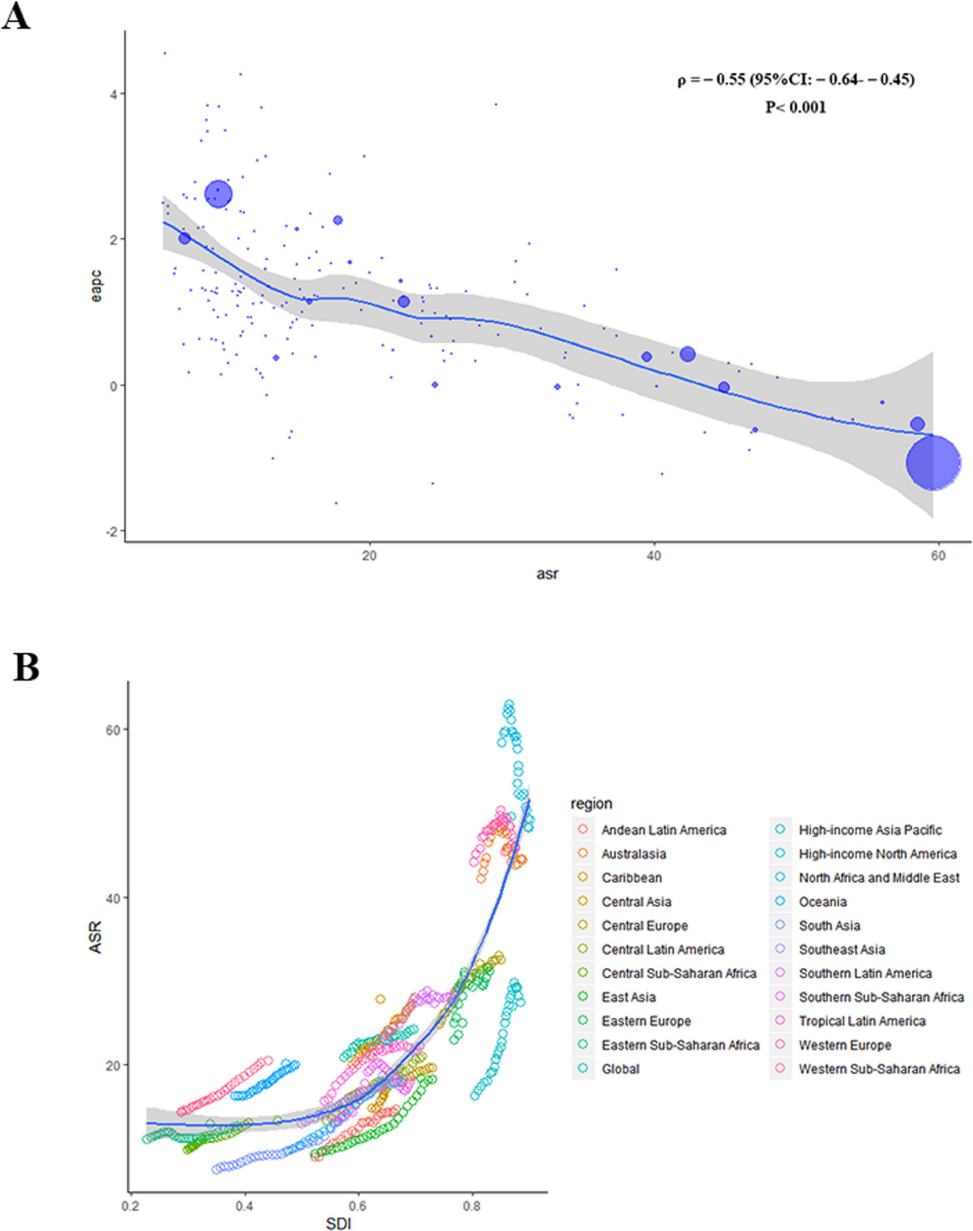
The correlation of EAPC and breast cancer ASR in 1990 (**a**) and the correlation of breast cancer ASR in 2017 and SDI (**b**). **a** The size of circle is increased with breast cancer cases and one circle represents a specific country. The *ρ* indices and *P* value were derived from Pearson correlation analysis. **b** The blue line represents the average expected relationship between SDIs and ASRs for breast cancer based on values from all countries from 1990 to 2017. ASR, age-standardized rate; EAPC, estimated annual percentage change; SDI, social-demographic index

A significant negative relationship was found between EAPCs and ASRs (*ρ* = − 0.55, *p* < 0.001), suggesting that breast cancer cases increased more slowly in higher incident countries than in lower incident countries (Fig. 3a). And the relationship between ASRs and SDI for each of the 21 GBD regions is shown in Fig. 3b, the ASRs tend to be numerically bigger in higher SDI regions than that of lower SDI regions.

### Age-related incidence

**Fig. 4:**
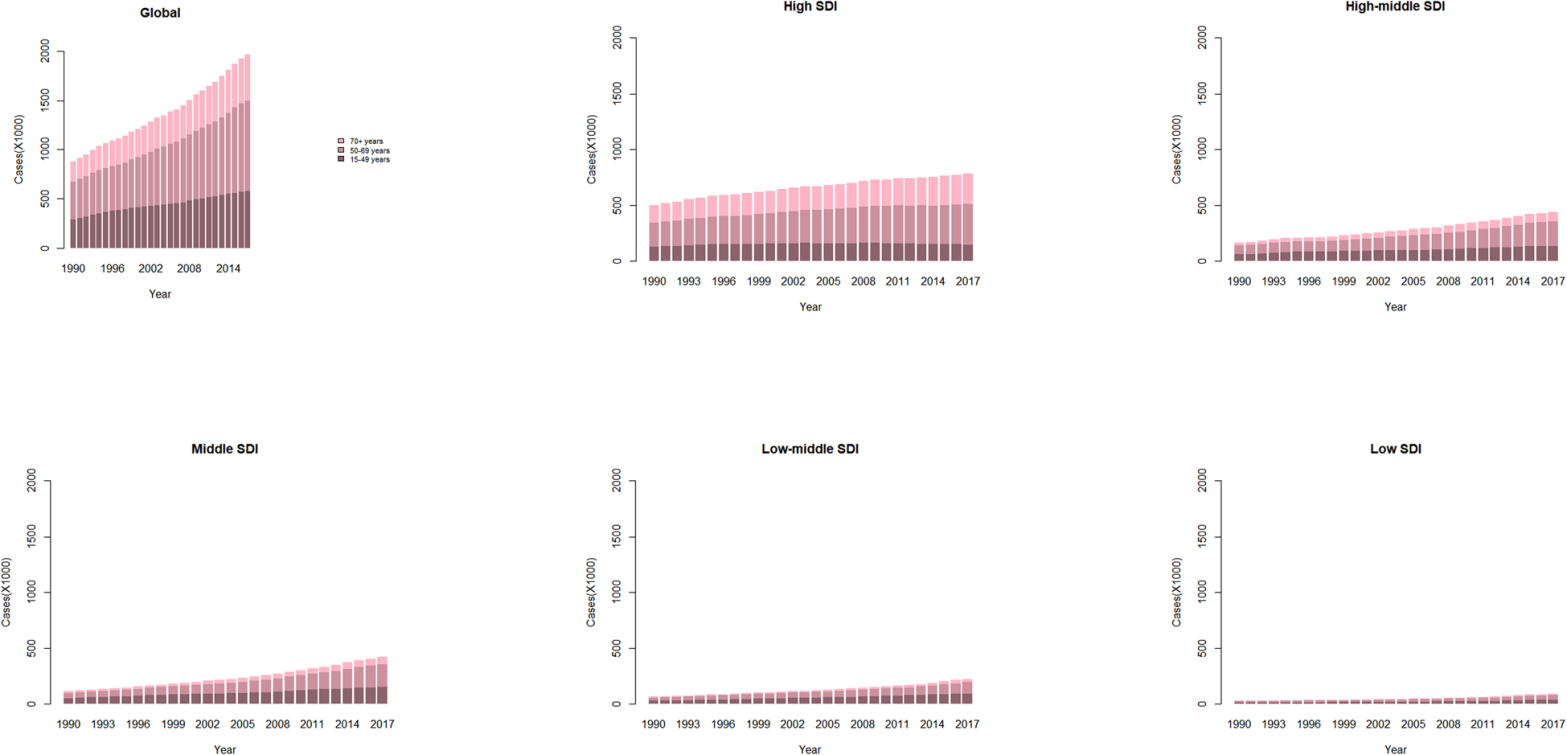
The proportion of the three age groups for breast cancer incident cases between 1990 and 2017 globally, and in high, high middle, middle, low middle, and low SDI quintiles. The populations were divided into three age groups: 15–49 years, 50–69 years, and 70+ years

Globally, breast cancer incidence in the 50–69-year age group was the highest among the three age groups (15–49 years, 50–69 years, and 70+ years). As shown in Fig. 4, the number of incident cases increased fastest in the low SDI quintile. Distinctly, the proportion of elderly breast cancer incident cases was the largest in the high SDI quintile and the smallest in the low SDI quintile.

As shown in Additional file 2: Figure S2a and b, the proportion of incidence in the three age groups remained stable between 1990 and 2017. Regionally, young breast cancer incidence was relatively higher in Oceania and Southeast Asia, and elderly breast cancer incidence was higher in Western Europe and the USA. However, young breast cancer incidence decreased, but elderly breast cancer incidence increased visibly in high-income Asia Pacific region.

### Risk factors attributable to breast cancer burden

**Fig. 5:**
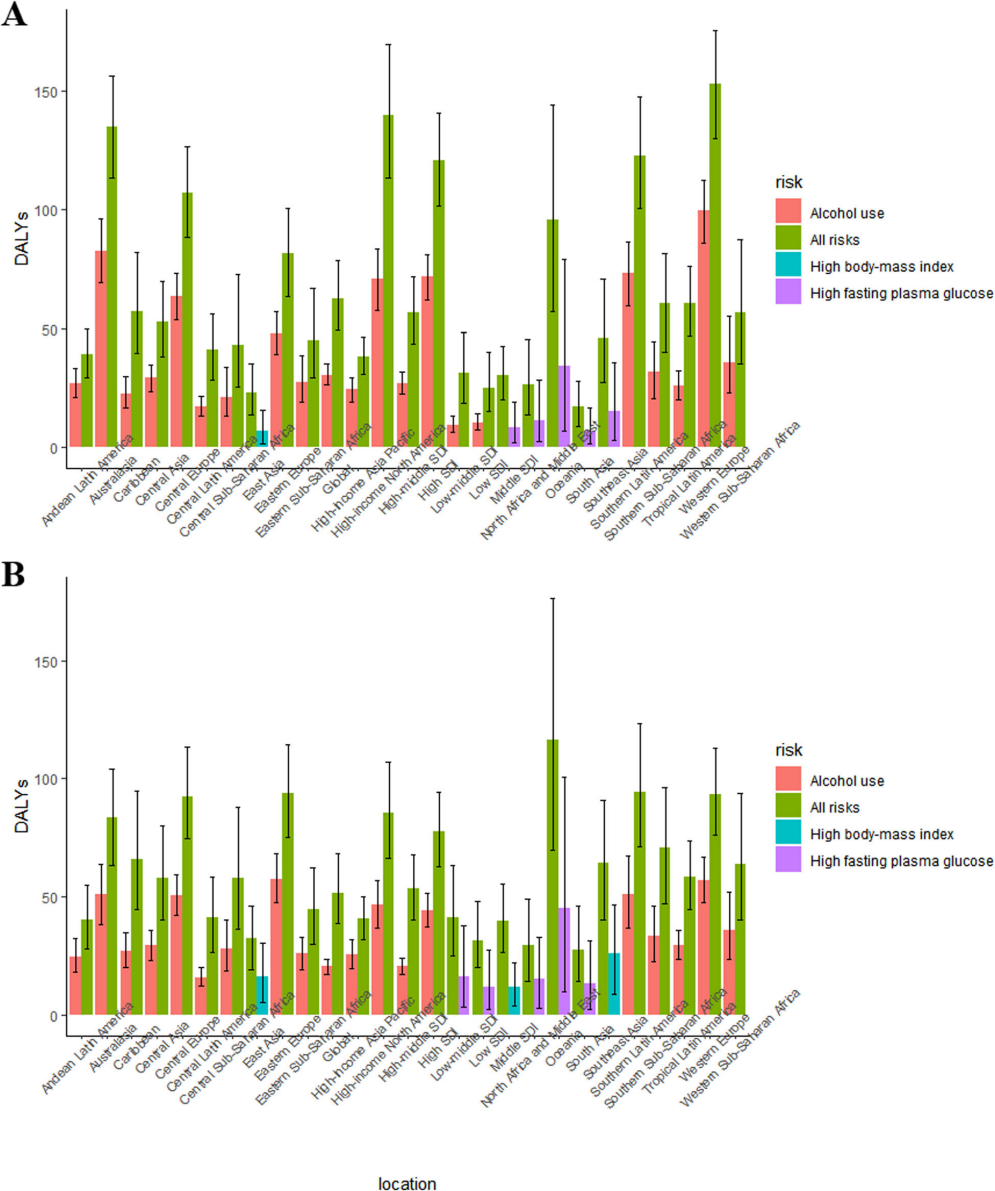
**a**, **b** The breast cancer DALYs attributable to risk factors compared in 1990 and 2017, both genders, globally and by region. Only the overall DALYs and the most pronounced one attributable to specific risk factors (alcohol use, high body mass index, and high fasting plasma glucose) were presented. DALY, disability-adjusted life year

Generally, alcohol use was the greatest distributor of DALYs in most GBD regions and other distributors included high body mass index (BMI) and high fasting plasma glucose. It was noteworthy that East Asia was the only region in which high BMI contributed the most to DALY cases in 1990. However, in 2017, high BMI also contributed to DALYs of Southeast Asia and middle SDI countries. In 1990, the regions in which high fasting plasma glucose contributed the most are the middle SDI countries (Fig. 5).

**Fig. 6:**
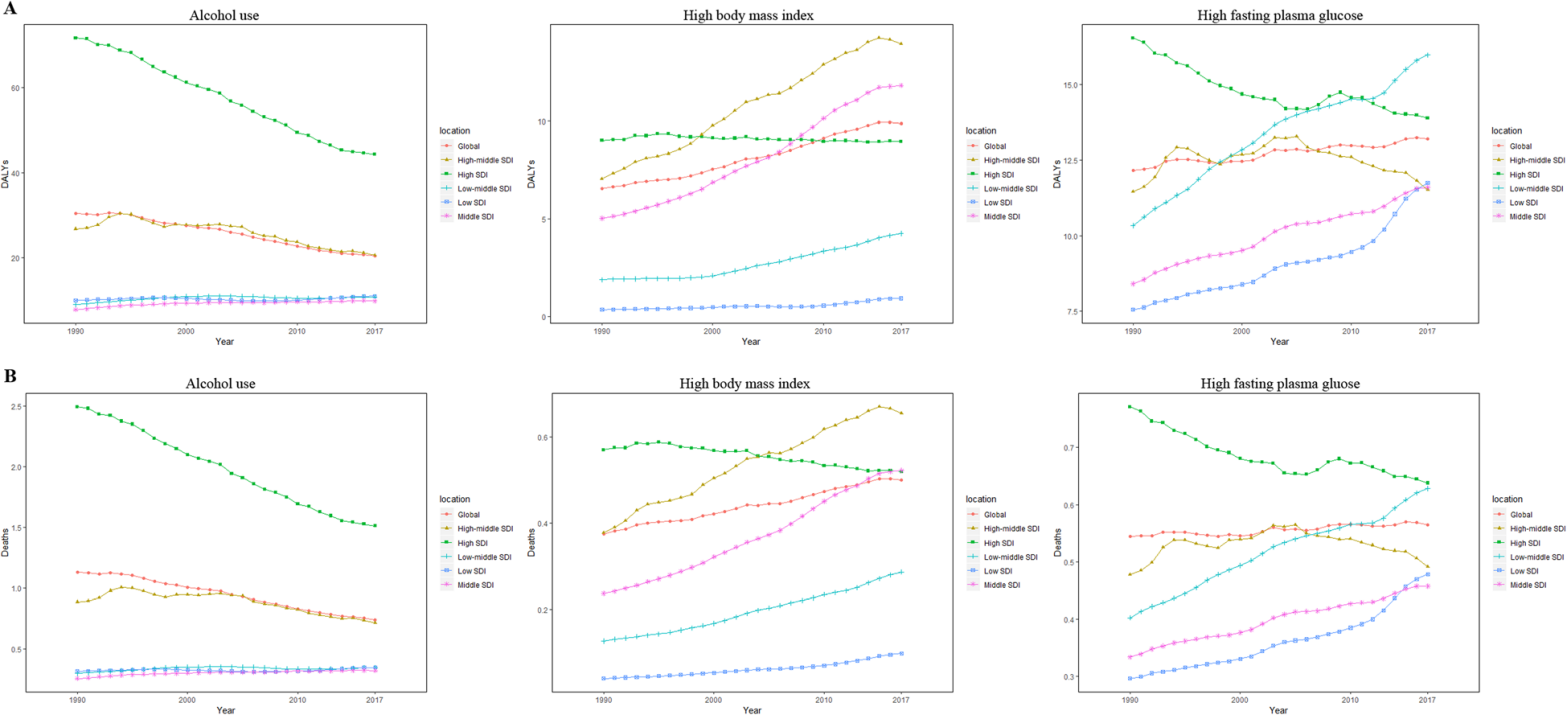
The age-standardized rates of DALYs (**a**) and deaths (**b**) attributable to alcohol use, high body mass index, and high fasting plasma glucose from 1990 to 2017, both sexes

Breast cancer DALYs attributable to alcohol use varied by gender and SDI quintiles in 2017. Of the 17,708,600 (95% UI = 16,899,498–18,674,972) global breast cancer DALYs, 9.43% (95% UI = 7.92–10.97%) was attributable to alcohol use, including 9.24% (95% UI = 7.76–10.76%) in females and 20.18% (95% UI = 15.37–25.05%) in males. Alcohol use-related breast cancer DALYs decreased globally between 1990 and 2017. On the contrary, alcohol attributed breast cancer DALYs increased in the low SDI, low middle SDI, and the middle SDI quintiles (Fig. 6a).

High body mass index led to 4.61% (95% UI = 1.52–8.83%) of global breast cancer DALYs in 2017 with an increasing trend between 1990 and 2017. Except the gentle trend of high BMI attributed DALYs in the high SDI quintile, the other quintiles showed increased trends, especially in the high middle SDI and the middle SDI countries (Fig. 6a).

In 2017, 6.07% of breast cancer DALYs was attributable to high fasting plasma glucose (95% UI = 1.15–13.53%) with a slight increase. In the high SDI quintile, the ASR has been decreasing since 1990, when it was the highest among the five quintiles. However, the rate was of great fluctuation between 1990 and 2017 while the low SDI, low middle SDI, and the middle SDI countries exhibited distinct increase trends (Fig. 6a).

The deaths attributable to risk factors mirrored the same pattern of attributable DALYs (Fig. 6b). With regard to risk factors, there was some difference in gender. In female breast cancer population, the global disease burden database indicated 6 risk factors of DALYs and deaths including alcohol use, high body mass index, high fasting plasma glucose, low physical activity, smoking, and second hand smoke. However, in males, the risk factors included alcohol use and second hand smoke, and the rate of contribution in 2017 was 20.18% (15.37–25.05%) and 1.51% (0.35–2.61%), respectively.

## Discussion

As far as we know, this GBD-based study reveals the most up-to-date trends and patterns of the incidence, mortality, and DALYs associated with breast cancer worldwide and the most relevant risk factors. Our analysis revealed that with the 1,960,681 new cases in 2017, the global incident cases of breast cancer increased by 123% between 1990 and 2017, but changes in ASIR (16% increasing) were less prominent. According to the global cancer burden 2016 [18], the changes in breast cancer incident cases were mainly attributable to population growth (12.4%) and aging (15.7%) rather than age-specific incidence rates (0.9%). Specifically, in the high SDI and the high middle SDI countries, the incident cases attributable to change of age-standardized incidence rate were close to none or even negative. On the contrary, changes in incidence rate contributed a lot in the middle SDI and the low middle SDI countries while in the low SDI quintile, incident cases due to population growth accounted for the largest proportion.

The trend of global age-standardized DALY rate was considerably varied in females and males, between 1990 and 2017. There was a gentle decline of age-standardized DALY rate in females, while the rate was increasing in males in the same study period. This discrepancy partially reflected the consequence of different risk factors between the genders. The age-standardized DALY rate also differed in the SDI quintiles. In the low SDI, low middle SDI, and the middle SDI countries, the rate was increasing both in males and females, while in the high middle SDI countries, the DALY rate had the similar global pattern and in the high SDI countries, the rate was declining in both the genders. The global number of breast cancer deaths increased substantially during the study period in both genders. This increase was consistent with the increase in breast cancer incident cases. Global ASDR declined since 1990 which was mainly due to the decrease in the high SDI and the high middle SDI quintiles, while the ASDR of other three quintiles slightly increased, between 1990 and 2017. Much of these geographic and gender disparities could be explained by heterogeneity in the prevalence of risk factors, with alcohol use being the chief factor.

Alcohol use is the most important of the risk factors contributing to breast cancer deaths and DALYs, though the attributable DALYs have been declining from 1990 to 2017. Consistently, the global daily prevalence of alcohol use declined significantly during the study period, which was more pronounced in the high and the high middle SDI countries [19]. This finding was consistent with the observed decrease in breast cancer ASDR and age-standardized DALY rate. The perniciousness of alcohol is instantiated both in the genetic level (enhancement of DNA damage, interference with mitochondrial function) and in the epigenetic level (affecting DNA methylation status and histone modification) [20]. Notably, there is a possible dose-response relationship between alcohol drinking and breast cancer [21].

Moreover, high body mass index and high fasting plasma glucose were also identified as potential risk factors attributable to breast cancer deaths and DALYs, which showed an increasing contribution trend of the two risk factors globally, especially in the high SDI and the high middle SDI quintiles. According to Hyuna Sung’s study [22], the most conspicuous obesity increase occurred among males in high-income Western countries and among females in Central Asia, the Middle East, and North Africa, which is considered to be caused by global food system changes promoting high-calorie, low-nutrient foods, accompanied by decreased physical activity. Increase of BMI is associated with increased breast cancer risk, and it is more risky in Asians, when compared with North Americans and Europeans. Though acceleration of national wealth has been in accordance with an increasing in body weight [23], prosperity is not always correlated with high body mass: obesity rate is quite low in high-income Asian Pacific countries, which is likely due to their traditional low-calorie dietary habits and physical activities such as daily walking [24, 25]. Nevertheless, obesity prevalence is extremely high in some low-income countries, such as some Pacific Island nations and Egypt [26].

It is worth mentioning that risk factors differed in the subtypes of breast cancer [27], for instance, high body mass index was associated with an increased risk of triple negative breast cancer, while low physical activity contributed to attributable risk of ER+/PR+ subtypes. On the one hand, this discrepancy possibly resulted from the conversion of androgen to estrogen in adipose tissue, which had a more severe influence on hormone receptor (HR)–positive breast cancer types [28]. On the other hand, leptin and other hormones could have exerted stimulating effects on HR–negative breast cancer cell proliferation, invasion, and angiogenesis, either directly or indirectly [29].

Policy-makers require country-specific information on the burden of different cancers to assess the impact of cancer control programs, benchmark progress in their nation, and allocate limited resources in their health care systems. Given the fact that existing data in many countries are of low quality or missing, the GBD study results can be used by stakeholders to study the trends of different diseases in their respective locations.

GBD studies provide high-quality estimates of global cancer burden, yet there exist several limitations. One inevitable limitation is the uncertainty of GBD estimates in cases in which actual data on disease burden are unavailable, and the GBD estimates fill the vacancies in this occasion. Besides, differences in data collecting and coding, as well as quality of data sources, remain inevitable in this analysis pattern. Moreover, the fluctuations in incidence and mortality rates may partly reflect the detection bias related to adjustments in screening protocols instead of real changes in age-specific rates.

## Conclusion

The global burden of breast cancer has been increasing continuously between 1990 and 2017, although in some SDI quintiles, the ASDR and age-standardized DALY rate has been declining. In recent years, disease reduction was observed in higher SDI regions while lower SDI regions had carried an incremental burden of breast cancer, and there may be a widening in disparities in the years to come. Consequently, steps against attributable risk factors should be taken to reduce breast cancer burden especially in lower SDI countries, to prevent acceleration of these disparities, because underdeveloped countries are affected to a greater degree by increases in health burden.

## Supplementary information

**Additional file 1: Figure S1.** Annual percent changes of age-standardized breast cancer incidence (A), death (B), and DALY (C) in 195 countries and territories between 2007 and 2017.

**Additional file 2: Figure S2.** The proportion of three age groups for breast cancer incident cases in both genders, globally and by region, contrasted in 1990(A) and 2017(B). The populations were divided into three age groups: 15-49 years, 50-69 years and 70+ years.

## Data Availability

The datasets supporting the conclusions of this article are included within the article.

## Abbreviations

ASDR: Age-standardized death rate
ASIR: Age-standardized incidence rate
ASR: Age-standardized rate
DALY: Disability-adjusted life year
EAPC: Estimated annual percentage change
GBD: Global burden of disease
HR: Hormone receptor
SDI: Social-demographic index
UI: Uncertainty interval
YLD: Year lived with disability
YLL: Year of life lost

## References

[1] Global, regional, and national incidence, prevalence, and years lived with disability for 354 diseases and injuries for 195 countries and territories, 1990-2017 (2018). a systematic analysis for the Global Burden of Disease Study 2017. Lancet (London, England).

[2] Giordano SH (2018). Breast cancer in men. New England Journal of Medicine..

[3] Collaboration GBoDC (2018). Global, regional, and national cancer incidence, mortality, years of life lost, years lived with disability, and disability-adjusted life-years for 29 cancer groups, 1990 to 2016: a systematic analysis for the Global Burden of Disease Study Global Burden of Cancer, 1990 to 2016Global Burden of Cancer, 1990 to 2016. JAMA oncology..

[4] Lin L, Yan L, Liu Y, Yuan F, Li H, Ni J (2019). Incidence and death in 29 cancer groups in 2017 and trend analysis from 1990 to 2017 from the Global Burden of Disease Study. J Hematol Oncol.

[5] Ginsburg O, Bray F, Coleman MP (2017). The global burden of women’s cancers: a grand challenge in global health. Lancet (London, England).

[6] Hashim MJ, Al-Shamsi FA, Al-Marzooqi NA, Al-Qasemi SS, Mokdad AH, Khan G (2018). Burden of breast cancer in the Arab world: findings from Global Burden of Disease, 2016. J Epidemiol Glob Health..

[7] The burden of cancers and their variations across the states of India (2018). the Global Burden of Disease Study 1990-2016. The Lancet. Oncology.

[8] Cazap E (2018). Breast cancer in Latin America: a map of the disease in the region. American Society of Clinical Oncology educational book. American Society of Clinical Oncology. Annual Meeting.

[9] Begum M, Lewison G, Jassem J (2018). Mapping cancer research across Central and Eastern Europe, the Russian Federation and Central Asia: implications for future national cancer control planning. European journal of cancer (Oxford, England : 1990).

[10] Global, regional, and national incidence, prevalence, and years lived with disability for 328 diseases and injuries for 195 countries, 1990-2016 (2017). a systematic analysis for the Global Burden of Disease Study 2016. Lancet (London, England).

[11] Foreman KJ, Lozano R, Lopez AD, Murray CJ (2012). Modeling causes of death: an integrated approach using CODEm. Popul Health Metr.

[12] Global, regional, and national under-5 mortality, adult mortality, age-specific mortality, and life expectancy, 1970-2016 (2017). a systematic analysis for the Global Burden of Disease Study 2016. Lancet (London, England).

[13] Zhou L, Deng Y, Li N (2019). Global, regional, and national burden of hodgkin lymphoma from 1990 to 2017: estimates from the 2017 Global Burden of Disease Study. Journal of hematology & oncology.

[14] Murray CJ, Lopez AD (1999). On the comparable quantification of health risks: lessons from the Global Burden of Disease Study. Epidemiology (Cambridge, Mass.).

[15] Hung GY, Horng JL, Yen HJ, Lee CY, Lin LY (2015). Changing incidence patterns of hepatocellular carcinoma among age groups in Taiwan. Journal of hepatology..

[16] Gao S, Yang WS, Bray F (2012). Declining rates of hepatocellular carcinoma in urban Shanghai: incidence trends in 1976-2005. Eur J Epidemiol.

[17] Hankey BF, Ries LA, Kosary CL (2000). Partitioning linear trends in age-adjusted rates. Cancer causes & control : CCC..

[18] Collaboration GBoDC (2017). Global, regional, and national cancer incidence, mortality, years of life lost, years lived with disability, and disability-adjusted life-years for 32 cancer groups, 1990 to 2015: a systematic analysis for the Global Burden of Disease Study Global Burden of Cancer 2015Global Burden of Cancer 2015. JAMA oncology..

[19] Zakhari S, Hoek JB (2015). Alcohol and breast cancer: reconciling epidemiological and molecular data. Advances in experimental medicine and biology.

[20] Zakhari S, Hoek JB. Epidemiology of moderate alcohol consumption and breast cancer: association or causation? Cancers. 2018;10: undefined. 10.3390/cancers10100349 PMC6210419 30249004

[21] Seitz HK, Pelucchi C, Bagnardi V, La Vecchia C (2012). Epidemiology and pathophysiology of alcohol and breast cancer: update 2012. Alcohol and alcoholism (Oxford, Oxfordshire).

[22] Sung H, Siegel RL, Torre LA (2019). Global patterns in excess body weight and the associated cancer burden. CA: a cancer journal for clinicians.

[23] Masood Mohd, Reidpath Daniel D. (2017). Effect of national wealth on BMI: An analysis of 206,266 individuals in 70 low-, middle- and high-income countries. PLOS ONE.

[24] Lee MJ, Popkin BM, Kim S (2002). The unique aspects of the nutrition transition in South Korea: the retention of healthful elements in their traditional diet. Public health nutrition..

[25] Mori N, Armada F, Willcox DC (2012). Walking to school in Japan and childhood obesity prevention: new lessons from an old policy. American journal of public health.

[26] Aitsi-Selmi A, Chandola T, Friel S, Nouraei R, Shipley MJ, Marmot MG (2012). Interaction between education and household wealth on the risk of obesity in women in Egypt. PLoS One.

[27] Turkoz FP, Solak M, Petekkaya I (2013). Association between common risk factors and molecular subtypes in breast cancer patients. Breast (Edinburgh, Scotland).

[28] Althuis MD, Fergenbaum JH, Garcia-Closas M, Brinton LA, Madigan MP, Sherman ME (2004). Etiology of hormone receptor-defined breast cancer: a systematic review of the literature. Cancer epidemiology, biomarkers & prevention : a publication of the American Association for Cancer Research, cosponsored by the American Society of Preventive Oncology.

[29] Vona-Davis L, Rose DP (2007). Adipokines as endocrine, paracrine, and autocrine factors in breast cancer risk and progression. Endocrine-related cancer.

