# Supplementary figures and images for: Global burden of breast cancer and attributable risk factors in 195 countries and territories, from 1990 to 2017: results from the Global Burden of Disease Study 2017
Source: J Hematol Oncol. 2019 Dec 21;12:140. doi: 10.1186/s13045-019-0828-0 (PMC6925497; doi:10.1186/s13045-019-0828-0)

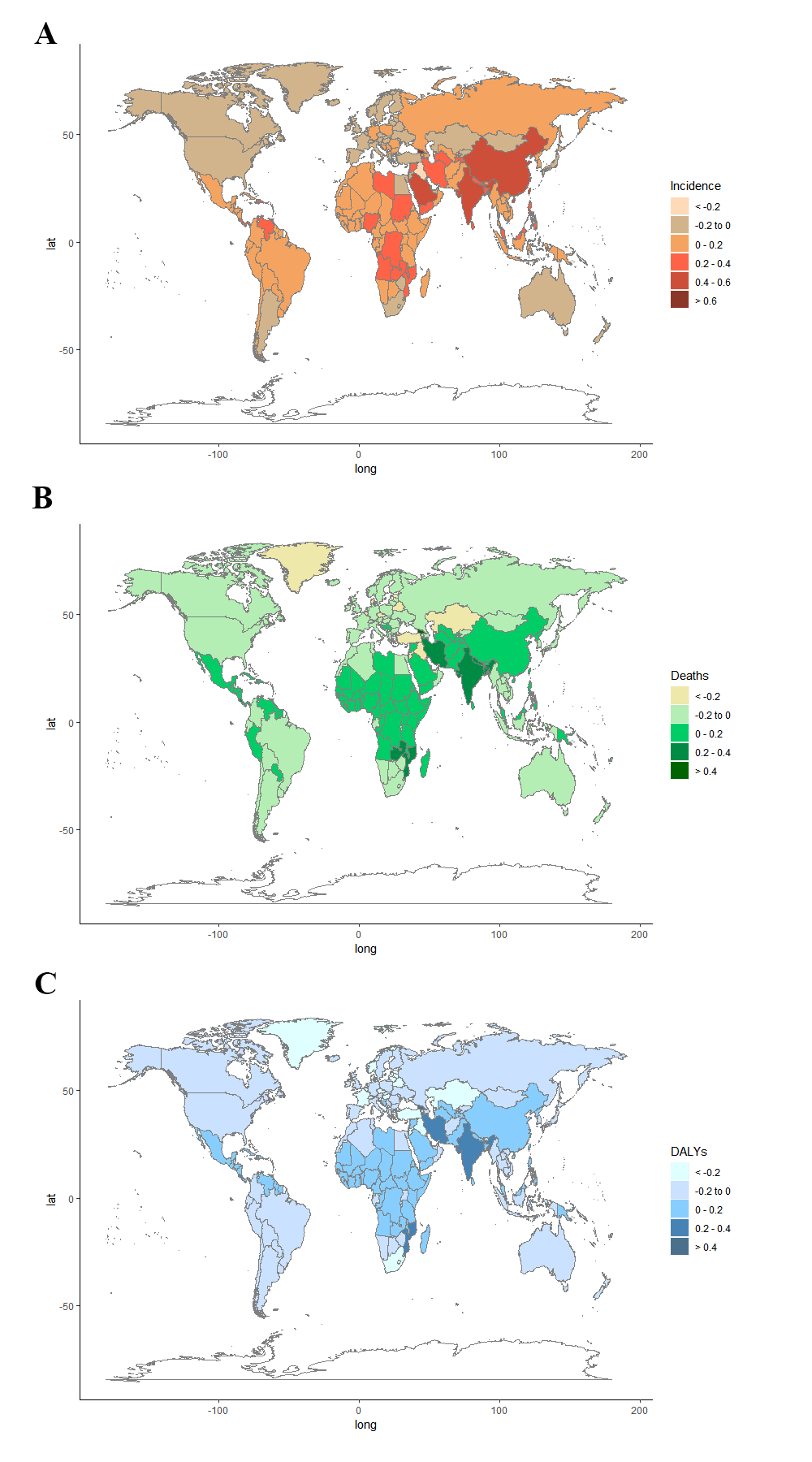

Supplement: Supplementary file 1 — Additional file 1: Figure S1. Annual percent changes of age-standardized breast cancer incidence (A), death (B), and DALY (C) in 195 countries and territories between 2007 and 2017. [file 13045_2019_828_MOESM1_ESM.tif]

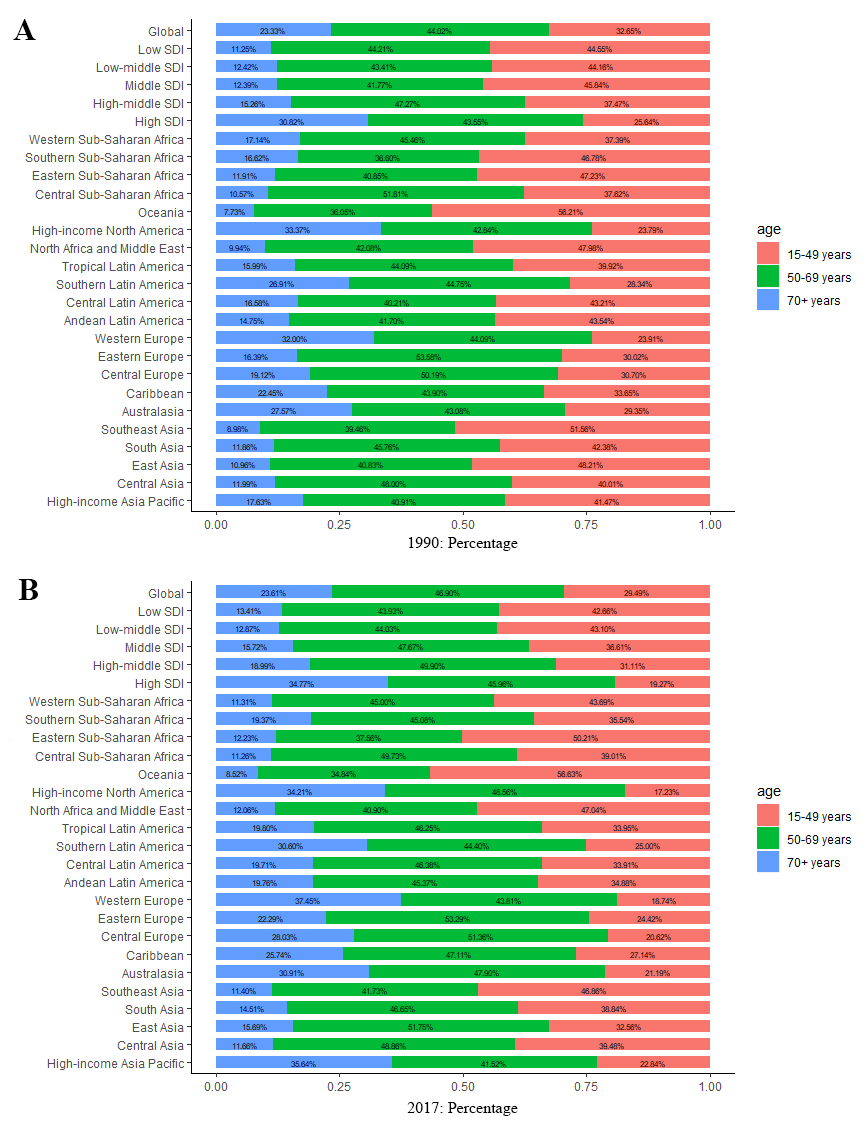

Supplement: Supplementary file 2 — Additional file 2: Figure S2. The proportion of three age groups for breast cancer incident cases in both genders, globally and by region, contrasted in 1990(A) and 2017(B). The populations were divided into three age groups: 15-49 years, 50-69 years and 70+ years. [file 13045_2019_828_MOESM2_ESM.tif]
